# Evaluation of Skeletal Changes after Mandibular Setback Surgery Using the NM-Low Z Plasty Technique in Skeletal Class III Patients

**DOI:** 10.1055/s-0042-1749364

**Published:** 2022-07-04

**Authors:** Sarunpong Imampai, Siripatra Patchanee, Awiruth Klaisiri, Narissaporn Chaiprakit

**Affiliations:** 1 Division of Orthodontics, Faculty of Dentistry, Thammasat University, Pathum Thani, Thailand; 2 Thammasat University Research Unit in Mineralized Tissue Reconstruction, Thammasat University, Pathum Thani, Thailand; 3 Division of Restorative Dentistry, Faculty of Dentistry, Thammasat University, Pathum Thani, Thailand; 4 Thammasat University Research Unit in Restorative and Esthetic Dentistry, Thammasat University, Pathum Thani, Thailand; 5 Division of Oral and Maxillofacial Surgery, Faculty of Dentistry, Thammasat University, Pathum Thani, Thailand

**Keywords:** malocclusion, mandibular setback, orthodontics, orthognathic surgery, skeletal class III, skeletal relapse

## Abstract

**Objective** The study's objective was to evaluate skeletal changes in 38 skeletal class III patients following mandibular setback surgery using NM-Low Z plasty.

**Materials and Methods** Thirty-eight skeletal class III patients (ANB angle lower than 0) who underwent the NM-Low Z plasty technique for surgical mandibular setback procedure at Thammasat University Hospital between January 2017 and March 2020 were included in the study: 29 patients had two jaw surgeries, and 9 patients had one jaw surgery. An additional 14 patients had genioplasty. Three lateral cephalograms were traced and digitized with Dolphin Imaging software: T0, T1, and T2. The distance between the B-point and the SN7 perpendicular line defined immediate changes after surgery (T1-T0) and stability after surgery (T2-T1). The reliability test included 6 cephalograms retraced after 2-week interval. At point B, the principal result was horizontal movement forward.

**Statistical Analysis** The analysis used paired *t* -tests.

**Results** The mean mandibular setback was 9.78 mm, and the mean skeletal relapse was 2.61 mm, or 26.69%. Statistical analysis showed postoperative differences ( *p* < 0.05). Vertical measurement in B-SN7 reduced immediately and postoperatively.

**Conclusion** Postoperatively, the mandible relapsed significantly forward and upward. Rotational relapse is a concern with NM-Low Z plasty in hypo- **/** normodivergent patients.

## Introduction

Skeletal class III is a skeletal discrepancy featured by mandibular prognathism with or without maxillary retrognathism that is associated with a concave facial profile. Southeast Asian populations show the highest prevalence rate (15.08%) of class III malocclusion. 1

Possible treatments to correct skeletal class III malocclusion are growth modification in growing patients, dental camouflage by orthodontic treatment, and orthognathic surgery combined with orthodontic treatment in nongrowing patients. Mandibular setback surgery can be performed to move the mandible posteriorly and rotate and move it downward **/** upward anteriorly. The most popular surgical method to move the mandible posteriorly is bilateral sagittal split osteotomy (BSSO). Obwegeser and Trauner were the first to propose this surgery in 1957. 2 Subsequently, many modifications were proposed, such as those by Dalpont, Hunsuck, and Epker. 2 3 In 2016, Tangarturonrasme and Sununliganon introduced the BSSO low Z plasty technique (Prasan's modification). This method allows the mandible to be set posteriorly to a larger extent and reduces possible complications. 4 With this technique, skeletal stability and satisfactory results can be achieved. Chaiprakit et al then proposed the NM-Low Z plasty technique, 5 which removes bony interference on the internal surface of the proximal segment. Thus, the surgeon can more easily conduct mandible surgery in three dimensions. Currently, this technique is one of the surgical procedures used in our center.

Cephalometric study is one of the investigating tools for analysis of facial skeletal, dental, and soft tissue profile of multistage treatment. 6 7 Skeletal relapse or skeletal instability is considered an undesired result of orthognathic treatment. The cause for this relapse is thought to be multifactorial. One of the contributions to skeletal relapse is the surgical technique. Kim et al 8 proved that BSSO and concomitant mandibular angle resection tended to influence postsurgical skeletal instability. Various modifications of conventional BSSO can lower the tension of pterygomasseteric slings and achieve more clinical aesthetic results in mandibular prognathism patients with squared faces and prominent mandibular angles. Kim et al 9 also reported a procedure to reduce postsurgical relapse after mandibular setback using BSSO. They compared a technique including intended osteotomy of the distal segment's posterior portion to the conventional one. This distal osteotomy technique has been used since 1994. Without the distal osteotomy technique, the distal segment protrudes into the gonial region as the mandible is set back. Consequently, lengthening the pterygoid muscle causes relapse and reduces the posterior pharyngeal air space.

Although NM-Low Z plasty allows for less bony interference, which results in less bony contact, there is no report regarding skeletal stability following mandibular setback surgery. The aim of this research was to evaluate skeletal changes following mandibular setback surgery performed with the NM-Low Z plasty technique in skeletal class III patients.

## Materials and Methods

This study followed the Declaration of Helsinki guidelines regarding medical protocol and ethics, and the protocol was approved by the Ethics Committee of Thammasat University (119/2563).

Thirty-eight skeletal class III patients (ANB angle lower than 0) who underwent the NM-Low Z plasty technique for surgical mandibular setback procedure at Thammasat University Hospital between January 2017 and March 2020 were included in the study: 29 patients had two jaw surgeries, and 9 patients had one jaw surgery. An additional 14 patients had genioplasty ( Table 1 ). The same skilled surgeon conducted all of the orthognathic operations. The NM-Low Z plasty technique was followed to the protocol described in previous case report. 5 Inclusion criteria were: a complete series of identifiable lateral cephalograms and confirmation of growth completion by a cervical vertebral maturation status of CS6. 10 Patients with craniofacial anomalies or facial bone fractures and incomplete diagnostic data were excluded from the study.

**Table 1:** Demographic variables of the sample

Demographic variables ( *n* = 38)	
Age (y), mean ± SD (range)	24.8 ± 4.3 (16–37)
Women, % ( *n* )	65 (25)
With two jaws surgery, % ( *n* )	76 (29)
With one jaw surgery, % ( *n* )	24 (9)
With genioplasty, % ( *n* )	36.8 (14)

Abbreviation: SD, standard deviation.

**Fig. 1:**
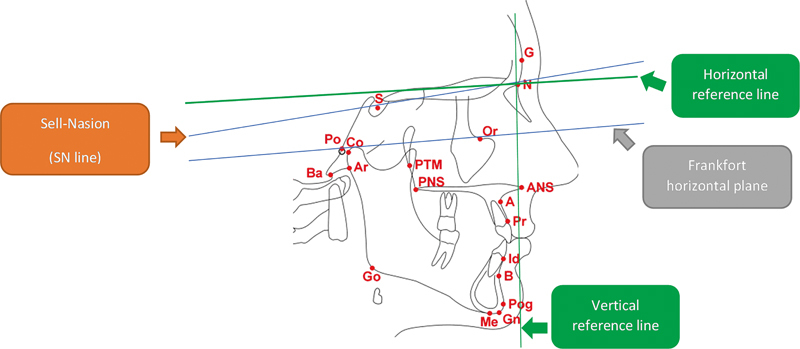
Reference points and reference lines used in cephalometric analysis.

A set of three standardized lateral cephalograms (T0: prior to surgery, T1: immediately following surgery, T2: 6 to 12 months after surgery) were obtained from patients. All cephalograms were digitized, traced, and measured into variables by one observer using Dolphin Imaging software, the reliability of which for both hard and soft tissue points equaled those traced by manual landmark plotting. 11 12 13 14 Moreover, problems with time consumption and various errors due to improper tracing, measurement, and calculation were diminished using computerized cephalometric software. The mean skeletal changes were defined in the variables at different stages: immediate postsurgical changes (T1-T0) and postsurgical stability (T2-T1). Reference lines were constructed for linear measurements. The vertical reference line was described as a line perpendicular to the line 7 degrees below the sella-nasion (SN) line. A horizontal reference line was drawn between the cephalometric marks porion (Po) and orbitale (Or), as shown in Table 2 . 15 Skeletal relapse was defined as forward movement in the horizontal direction at the deepest midline point on the bony curvature of the anterior mandible between the infradental and pogonion (point B) ( Fig. 1 ).

**Table 2:** Definition of the variables utilized in the study

Measurement	Definition
B-SN7perp (mm)	The perpendicular distance from B point to the line below 7° to the SN line
Pog-SN7perp (mm)	The perpendicular distance from pogonion to the line below 7° to the SN line
SNB (degrees)	The angle between the SN line and NB line
ANB (degrees)	The difference between angles SNA and SNB
Me-SN7 (mm)	The distance from Me to the line below 7° to the SN line
B-SN7 (mm)	The distance from B point to the line below 7° to the SN line
SN-GoGn (degrees)	The angle between the SN line and mandibular plane
IMPA (degrees)	The angle between line of lower incisor axis and mandibular plane

Abbreviations: IMPA, incisor mandibular plane angle; SN, sella-nasion.

To analyze the reliability test, 6 films from the 38 patients were randomly selected. At 2-week intervals, the cephalograms were retraced and measured, and the Dahlberg formula was used to compare linear measurements between two time periods.

(FIG), where *D* is the difference between two measurements and *N* denotes the number of double determinations. 16

**Fig. 2:**
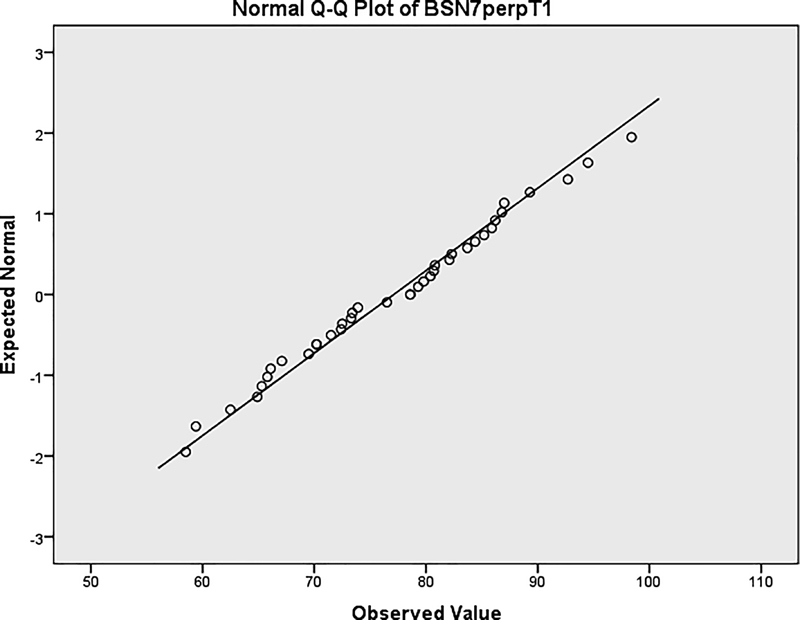
Normal Q-Q plot of horizontal measurement.

**Fig. 3:**
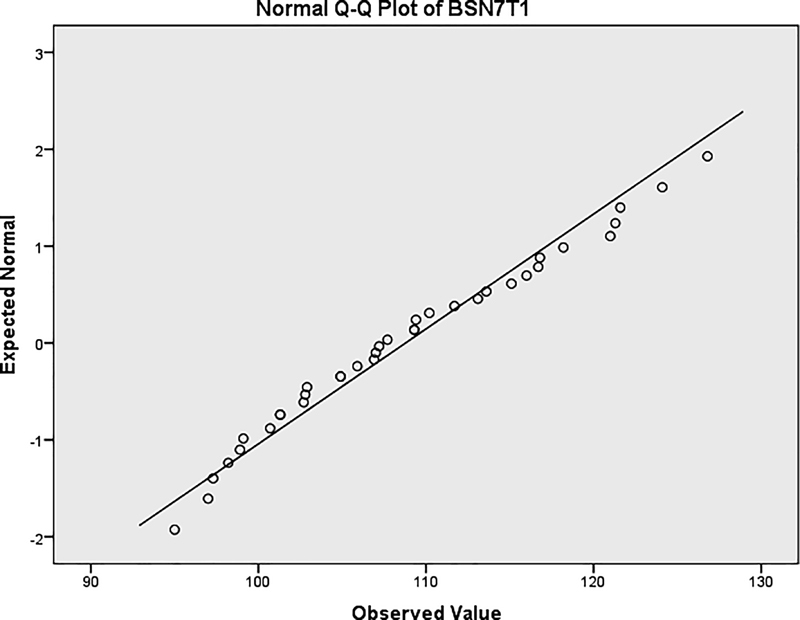
Normal Q-Q plot of vertical measurement.

The distribution of skeletal changes at point B was examined. The statistics showed that the measurement was normally distributed according to the Shapiro–Wilk test and Q-Q normality plot ( Figs. 2 and 3 , Table 3 ).

**Table 3:** Tests of normality of horizontal and vertical measurements

Normality tests						
	Kolmogorov–Smirnov			Shapiro–Wilk		
	Statistic	df	Significance	Statistic	df	Significance
BSN7perpT0	0.100	36	0.200	0.971	36	0.460
BSN7perpT1	0.084	36	0.200	0.982	36	0.817
BSN7perpT2	0.107	36	0.200	0.974	36	0.558
BSN7T0	0.082	36	0.200	0.976	36	0.622
BSN7T1	0.090	36	0.200	0.967	36	0.343
BSN7T2	0.090	36	0.200	0.963	36	0.275

Abbreviations: df, degree of freedom; T0: prior to surgery, T1: immediately following surgery, T2: 6 to 12 months after surgery; 95% confidence interval.

Note: B-SN7perp indicates horizontal measurement; B-SN7 indicates vertical measurement.

To compare mean skeletal changes in variables at different stages of the NM-Low Z plasty technique, the paired *t* -test was performed. Two-sided *p* -values < 0.05 were considered significant. IBM SPSS Software Version 22 (International Business Machines Corporation, Armonk, New York, United States) was used to conduct this statistical study.

## Results

### Anteroposterior Skeletal Relationship

At point B, the average mandibular setback was 9.78 mm, and the average skeletal relapse was 2.61 mm in this group of patients, indicating a 26.69% skeletal relapse rate (2.61/9.78). The statistical analysis showed that the difference between the before surgery and immediate postsurgical changes was significant ( *p* < 0.05). Obviously, the mandible was significantly setback repositioned (B-SN7 perp, Pog-SN7 perp, SNB, *p* < 0.05), and significantly relapsed forward during the postsurgical period (B-SN7 perp, Pog-SN7 perp, SNB, *p* < 0.05). This indicates that the mandible has relapsed significantly forward during the postsurgical follow-up period.

### Vertical Skeletal Relationship

There was a trend of decrease in vertical dimension postsurgically. B point showed significant upward relapse (B-SN7, *p* < 0.05) after surgery and follow-up period. Since sagittal and vertical skeletal relationship, the mandible demonstrated a forward-upward rotation or autorotation.

### Dental Relationship

The incisor mandibular plane angle (IMPA) decreased because of autorotational counterclockwise movement of the mandible immediately after removal of the stabilization splint (IMPA, *p* < 0.05). The mandible seemed to relapse in the forward direction, and class III mechanics were used to prevent this situation. Consequently, the lower incisors were lingually inclined. During the postsurgical period, the lower incisors retroclined to maintain proper overjet, while the mandible had a tendency to relapse in the anterior position.

## Discussion

In patients with skeletal III relationship, the mandible was moved backward and inferiorly repositioned during mandibular setback surgery ( Table 4 ). After surgery, the mandible then moved in forward and superior directions.

**Table 4:** Comparison of skeletal and dental variables according to patients who underwent NM-Low Z plasty at various stages

Variables	Measurement	T0		T1		T2		*p*		
		Mean	SD	Mean	SD	Mean	SD	T1-T0	T2-T1	T2-T0
Horizontal skeletal variable	B-SN7perp (mm)	81.46	13.52	71.68	12.45	74.28	12.7	0.000 a	0.000 a	0.000 a
	Pog-SN7 perp (mm)	83.29	14.96	75.33	14.02	77.82	13.93	0.000 a	0.000 a	0.000 a
	SNB (degrees)	86.88	4.39	83.5	4.15	84.8	4.37	0.000 a	0.000 a	0.000 a
	ANB (degrees)	–3.65	2.65	2.09	1.68	1.17	2.04	0.000 a	0.000 a	0.000 a
	Me-SN7 (mm)	–128.04	15.75	–124.74	13.1	–122.78	14.4	0.017 a	0.077	0.001 a
Vertical skeletal variable	B-SN7 (mm)	105.32	12.99	102.1	10.95	99.96	11.57	0.009 a	0.041 a	0.000 a
	SN-GoGn (degrees)	31.82	5.94	32.58	6.5	32.08	7.12	0.255	0.228	0.726
Dental variable	IMPA (degrees)	85.88	7.6	79.32	6.56	79.46	7.75	0.000 a	0.839	0.000 a

Abbreviations: SD, standard deviation; T0, prior to surgery; T1, immediately following surgery; T2, 6 to 12 months following surgery.

Note: A paired *t* -test was conducted.

a *p* < 0.05 by paired *t* -test.

NM-Low Z plasty surgical osteotomy has several benefits over other modifications operated at mandible. By removing the medial side of the proximal segment, bony interference was reduced. This allows for more mandibular movement (set back or advancement) and minimizes the risk of interfering with essential structures such as the facial nerve and styloid process. Additionally, it minimizes the risk of compromising the pharyngeal airway space as compared with other techniques. 5

In this research, there was significant difference in vertical (B-SN7, *p* < 0.05) and horizontal skeletal variables (B-SN7 perp, *p* < 0.05) during the postsurgical period. These findings would explain why upward and forward movement of the mandible occurred during the postsurgical period. Our findings conformed to many previous studies, 17 18 19 which reported a greater horizontal relapse and more forward chin projection caused by bite closure after postsurgical orthodontic treatment. Additionally, Hsu et al reported that the most sagittal relapse in the postsurgical period contributed to the forward-upward rotation of the mandible. 20

There was a decrease in the vertical skeletal relationship in the postsurgical period. According to Ko et al, it was hypothesized that the vertical dimension was decreased because of autorotation of the mandible with the occlusal settling process of the posterior teeth in postsurgical orthodontic treatment, especially in cases with severe occlusal interferences. 17 Furthermore, Imerb et al stated that a high mandibular plane angle had less stability than a normal mandibular plane angle. 21

However, there were benefits in performing dental decompensation before surgery using the conventional approach. 22 23 From our finding that the lower incisors proclined after decompensation and retroclined after the postsurgical period, round-tripping tooth movement was observed, which was considered an adverse effect. This was in agreement with Capelozza et al, who reported dental decompensation to obtain more skeletal correction in patients with skeletal class III. 23

With rotational relapse (forward and upward movement of the mandible) immediately after surgery, there might be a benefit to patients with skeletal class III and hyperdivergent patterns (open-bite tendency) to decrease facial height. In contrast, hypo-/normodivergent patients must be treated with caution. Vertical control of overbite and anterior facial height are recommended in contemporary orthodontics. 24

In the finishing stage of comprehensive orthodontic treatment, normal overbite is one of the requirements before the removal of full fixed appliances. 25 To correct excessive overbite, two factors must be addressed: (1) the vertical relationship between upper lip and upper incisors and (2) anterior facial height. If there is an appropriate display of the upper frontal teeth while smiling, the position of the upper incisors needs to be maintained, and the overbite needs to be corrected by intruding the lower incisors instead of the upper incisors. If the display of the upper frontal teeth is excessive, intrusion of the upper incisors is suggested. In patient with a short facial height, extrusion of the lower posterior teeth is acceptable. If the patient has a long facial height, intruding the incisors is indicated. As with other issues, planning for impending events is critical while dealing with skeletal and dental relapses.

Various types of osteotomy modification, fixation, and postoperative protocol could explain the different relapse rate. Our study, NM-Low Z plasty, could confirmed that skeletal stability was comparable to the conventional Hunsuck-Epker procedure in the finite element analysis. 26 The results in this study were limited to NM-Low Z plasty surgical technique. Further study needs to be conducted to compare the amount and direction of skeletal changes with conventional surgical technique as well as biomechanical studies.

## Conclusion

Mandible was significantly relapsed in forward and upward direction following mandibular setback surgery using NM-Low Z plasty technique. Lower incisors were retroclined after surgery to compensate autorotational counterclockwise of mandible. Patient selection, overcorrection, and orthodontic mechanics to deal with relapse must all be considered.
